# Development and Validation of a Discharge Readiness Scale for First Allo‐HSCT Patients

**DOI:** 10.1155/jonm/2080456

**Published:** 2026-08-16

**Authors:** Jiao Zhao, Yuanyuan Feng, Jiulian Yuan, Yanmin Zhao, Yan Chen, Mengxiao Chen, Bei Zhang, Ying Zhu

**Affiliations:** ^1^ Bone Marrow Transplantation Center, The Affiliated Hospital of Xuzhou Medical University, Xuzhou, Jiangsu, China, xzmc.edu.cn; ^2^ The Second Affiliated Hospital of Xuzhou Medical University, Xuzhou, Jiangsu, China, xzmc.edu.cn

**Keywords:** allogeneic hematopoietic stem cell transplantation, discharge readiness, nursing management, reliability, scale development, validity

## Abstract

**Aims:**

To develop and validate a disease‐specific discharge readiness scale for patients undergoing the first allogeneic hematopoietic stem cell transplantation (allo‐HSCT).

**Background:**

Patients undergoing allo‐HSCT face unique postdischarge challenges, but no disease‐specific assessment tool exists to guide nursing management.

**Methods:**

A cross‐sectional study was conducted with 163 patients. Scale development included literature review, expert consultation, and psychometric testing (item analysis, exploratory factor analysis, confirmatory factor analysis, and reliability testing).

**Results:**

The final 24‐item scale comprised four dimensions: Physiological Stability; Psychological Competence; Information Knowledge and Coping Ability; and Social Support. The scale demonstrated excellent psychometric properties, with a scale‐level content validity index of 0.92, explaining 83.488% of the total variance, a Cronbach’s α of 0.858, and a test–retest reliability coefficient of 0.965.

**Conclusions:**

The scale is a reliable and valid tool for assessing discharge readiness in this patient group.

**Implications for Nursing Management:**

This scale enables nursing managers to identify patients at risk, allocate resources effectively, and deliver targeted discharge interventions to improve postdischarge outcomes.

## 1. Introduction

Allogeneic hematopoietic stem cell transplantation (allo‐HSCT) is the primary curative treatment for various hematological malignancies. With the advancement of transplantation technology and the improvement of clinical nursing level, the 10‐year survival rate of allo‐HSCT patients has reached 80%–92% [1–3]. However, allo‐HSCT patients face a long immune reconstitution cycle after transplantation, accompanied by potential complications such as graft‐versus‐host disease (GVHD), long‐term medication dependence, and body image changes, which bring great challenges to postdischarge home rehabilitation and nursing management practice.

Discharge readiness, first proposed by Fenwick [4] in 1979, refers to patients’ comprehensive ability to leave the hospital, return to family, and continue the rehabilitation process, which is closely related to postdischarge prognosis, readmission rate, and quality of life. At present, clinical practice mostly adopts generic discharge readiness scales, which fail to accurately reflect the specific rehabilitation needs of allo‐HSCT patients due to the particularity of the disease and treatment process, leading to difficulties in precise nursing management. In addition, there are significant differences between allo‐HSCT and autologous hematopoietic stem cell transplantation in preconditioning regimens, complication risks, treatment cycles, and medical costs, underscoring the need for a disease‐specific discharge readiness scale for this population. To align with national priorities for improving care quality and reducing avoidable readmissions, the “Healthy China 2030” initiative has set a requirement to control the average length of hospital stay within 10.2 days. In this context, allo‐HSCT patients are often discharged before completing rehabilitation, making accurate discharge readiness assessment a critical component of nursing management [5, 6]. While generic discharge readiness tools such as the Readiness for Hospital Discharge Scale (RHDS) are widely used in general populations, they lack disease‐specific items addressing allo‐HSCT‐related concerns, including GVHD monitoring and long‐term immunosuppressive medication dependence. This gap highlights the need for a tailored scale to support targeted nursing management in this vulnerable patient group.

Existing studies have shown that disease‐specific assessment tools can effectively improve the efficiency of nursing management and the relevance of interventions [7]. Based on Galvin’s [8] operational definition of discharge readiness and the core requirements of clinical nursing management, this study developed a discharge readiness assessment scale for patients undergoing the first allo‐HSCT through literature review, expert consultation, and empirical investigation, and comprehensively tested its reliability and validity. The primary objectives of this study were twofold: (1) to develop a disease‐specific discharge readiness scale tailored for patients undergoing their first allo‐HSCT; (2) to validate the psychometric properties of the newly developed scale, including reliability and validity. This research intends to deliver a dedicated discharge evaluation tool specifically for allo‐HSCT patients and offer empirical evidence to facilitate targeted post‐transplant nursing plans, reasonable deployment of medical resources, and improved postdischarge rehabilitation quality.

## 2. Methods

### 2.1. Establishment of the Research Group

The research group consisted of 1 head nurse from the Hematopoietic Stem Cell Transplantation Center, 3 postgraduate students majoring in nursing, and 5 clinical nurses with more than 5 years of working experience and the title of Nurse Specialist. The head nurse and one postgraduate student were responsible for the overall research design, scale development guidance, and expert consultation; two postgraduate students conducted literature retrieval, data entry, and sorting; and the five clinical nurses performed questionnaire distribution and collection and were trained on standard operating procedures for data collection. The head nurse and Postgraduate P1 were responsible for the overall research design, technical guidance in the scale development process, and screening and contact of consulting experts. Postgraduates P2 and P3 were in charge of data entry and sorting. The 5 clinical nurses undertook questionnaire distribution and collection. Postgraduates P1 and P2 were responsible for literature retrieval and analysis and training the 5 nurses on the standard operating procedures and key aspects of the questionnaire investigation.

### 2.2. Construction of the Initial Scale Item Pool

Based on Galvin’s [8] operational definition of discharge readiness and the practical needs of nursing management, the research group initially determined four core dimensions of the scale: Physiological Stability (good functional status and home self‐care ability), Psychological Competence (sufficient confidence to cope with discharge), Information Knowledge and Coping Ability (knowledge and skills to handle common postdischarge problems), and Social Support (support to meet various needs after discharge).

For literature retrieval, Chinese databases including CNKI, Wanfang Database, VIP Chinese Science and Technology Periodical Database, and China Biomedical Literature Database (CBM) were searched with the subject terms of “stem cell transplantation,” “allogeneic transplantation,” “discharge preparation,” “discharge planning,” and “nursing management.” Foreign databases, including PubMed, Web of Science, Cochrane Library, and Journal of Nursing Management, were searched by two independent researchers with the subject terms of “stem cell transplantation,” “allogeneic transplantation,” “discharge readiness,” “scale development,” and “nursing management.” The retrieval time was from the establishment of each database to September 30, 2022. Conference abstracts were excluded, and a total of 95 literature references were screened, with 5 literature references included for reference [8–12].

Referring to the generic RHDS, the Chinese version of RHDS revised by Zhao et al. [7], the short form of RHDS developed by Lin et al. [13], and the nursing management‐oriented scale development framework proposed by Weiss et al. [14], combined with expert group discussion and clinical nursing management practice of allo‐HSCT, the initial item pool was constructed with 39 items across the 4 aforementioned dimensions (6 items from generic scales, 8 items from reference literature, and 25 items from expert group discussion and nursing management practice).

### 2.3. Delphi Expert Consultation

#### 2.3.1. Selection of Experts

Purposive sampling was adopted to select 23 experts engaged in the specialized clinical medicine, specialized clinical nursing, or nursing management of hematological diseases. The inclusion criteria were as follows: (1) having at least 8 years of professional working experience in hematological diseases or nursing management; (2) holding the professional title of intermediate or above; (3) having a bachelor’s degree or above; (4) being rigorous in academic research; (5) voluntarily participating in this study and being able to complete the consultation questionnaire on time. The exclusion criterion was failure to return the questionnaire within the specified time. A total of 23 experts were invited to participate in the Delphi consultation. Of these, 22 experts completed both rounds of the survey, resulting in a response rate of 95.7%.

#### 2.3.2. Implementation of Expert Consultation

Two rounds of Delphi expert consultation were conducted via the WeChat Questionnaire Star platform from January 2023 to March 2023, with each round lasting for 2 weeks. The 5‐point Likert scale was used for experts to evaluate the importance and nursing management applicability of each item (1 point = completely inconsistent/inapplicable, 5 points = completely consistent/applicable). The item deletion criteria were set as follows [15]: the average importance score < 4 points, the average applicability score < 4 points, the coefficient of variation > 0.25, or the item‐level content validity index (I‐CVI) < 0.78. Items meeting two or more exclusion criteria or deemed clinically inconsistent were deleted after collective discussion. For example, Item 3 was removed due to ambiguity in describing patients’ self‐care behaviors; Item 9 was deleted for being overly broad and lacking operational clarity; Items 14, 15, and 16 were excluded due to implied inclusion relationships with other items; and Items 37 and 39 were removed as they were inconsistent with the general care model for allo‐HSCT patients. The consultation was repeated until the experts′ opinions reached a consensus.

### 2.4. Presurvey

This study was approved by the Ethics Committee of a Grade III Class A hospital in Xuzhou (Approval No. XYFY2023‐KL215‐01). A presurvey was conducted in April 2023 among 20 patients undergoing the first allo‐HSCT in the Bone Marrow Transplantation Center of the hospital’s Hematology Department using convenient sampling [15].

The inclusion criteria for participants were as follows: (1) aged ≥ 14 years; (2) diagnosed with hematological malignancies by bone marrow puncture; (3) undergoing the first allo‐HSCT; (4) having normal communication ability and being able to complete the questionnaire independently; (5) signing the informed consent form. The exclusion criteria were as follows: (1) complicated with other serious systemic diseases; (2) with mental diseases or intellectual defects; (3) with hearing or speech impairment; (4) participating in other clinical research projects at the same time.

In the presurvey, patients found the “very unimportant to very important’ anchors confusing” in the 5‐point Likert scale. This wording was revised to “completely inconsistent–completely consistent” to better align with patients’ understanding of self‐reported discharge readiness behaviors. After revising the scoring description to “completely inconsistent–completely consistent,” patients had a good understanding of the scale, and no items were modified. The average time to complete the questionnaire was 6–15 min. Notably, all participants in this presurvey (*n* = 20) were excluded from the subsequent formal survey, and no presurvey data were included in the final analysis to avoid potential bias.

### 2.5. Formal Survey

#### 2.5.1. Study Subjects

Convenient sampling was adopted to select patients undergoing the first allo‐HSCT who met the inclusion criteria in the Bone Marrow Transplantation Center of the Hematology Department of a Grade III Class A hospital in Xuzhou from May 2023 to June 2025. According to the formula for sample size estimation in biomedical research [16]: N = [Max (number of dimensions) × (5–10)] × [1+(10%–20%)], the required sample size was 154–336 cases. A total of 167 patients were initially enrolled, and 163 valid questionnaires were recovered, with an effective recovery rate of 97.60%. The actual sample size (*N* = 163) was close to the lower limit of the pre‐estimated range (154–336). To ensure data quality, a double‐verification mechanism was used. Questionnaires with regular answering patterns or logical contradictions were excluded, resulting in an invalid response rate of 2.4%.

#### 2.5.2. Research Tools

1. General information questionnaire: It included demographic and clinical characteristics of patients, such as age, gender, educational level, average monthly household income, marital status, employment status, medical expense payment method, disease type, donor source, presence of chronic diseases, driving time from home to hospital, length of stay in the transplantation ward, and presence of complications during transplantation.
2. The preliminary version of the discharge readiness assessment scale for patients undergoing the first allo‐HSCT: Developed after two rounds of Delphi expert consultation, it consisted of 28 items across 4 dimensions. The 5‐point Likert scale was used for scoring (1 point = completely inconsistent, 5 points = completely consistent), with a higher total score indicating a better discharge readiness of patients.

#### 2.5.3. Data Collection and Quality Control

Before the questionnaire distribution, the research group unified the interpretation standards of all scale items and conducted specialized training on nursing management‐related investigation skills for the 5 clinical nurses, including standardized explanation of research purposes, accurate recording of patient responses, and strict adherence to ethical requirements. Only nurses who passed the training assessment were allowed to participate in data collection.

Paper questionnaires were distributed by the trained research team members. After fully explaining the research purpose and obtaining informed consent from patients, patients completed the questionnaires independently, and the investigators answered their questions in a timely manner. For patients who could not complete the questionnaire independently, the investigators read the items one by one and recorded the answers accurately. After recovery, all questionnaires were checked by two independent researchers using a double‐verification mechanism: The first verification focused on completeness (supplementing missing items), and the second verification focused on accuracy (excluding questionnaires with regular answering patterns or logical contradictions) [16]. This two‐step quality control ensured the reliability of data and met the quality requirements of nursing management research.

#### 2.5.4. Item Analysis

Five comprehensive methods were used for item screening, and items that met at least 3 deletion criteria were eliminated [17]: (1) Critical value analysis: The total scores of the scale were sorted in descending order, the top 27% were defined as the high‐score group, and the bottom 27% were defined as the low‐score group; independent samples *t*‐test was conducted, and items with CR(t) < 3 or *p* > 0.05 were deleted. CR value < 3 indicates poor discrimination between high and low scorers. (2) Discrete trend analysis: Items with a standard deviation < 1 were deleted. This removes items with low response variability, which provide limited information about participants′ true readiness levels. (3) Correlation analysis: Items with a correlation coefficient *r* < 0.3 between the item score and the total scale score or *p* > 0.05 were deleted. This ensures that each item is sufficiently related to the overall construct of discharge readiness. (4) Factor analysis: Items with a common factor variance < 0.4 or a factor loading < 0.4 were deleted. This step identifies items that do not align well with the underlying factors of the scale. (5) Cronbach’s α coefficient analysis: Items that led to a significant increase in the Cronbach’s α of the total scale after deletion were eliminated. This removes items that reduce the scale’s internal consistency and reliability. Items meeting three or more of these elimination criteria were deleted after collective discussion by the research team.

**FIGURE 1:**
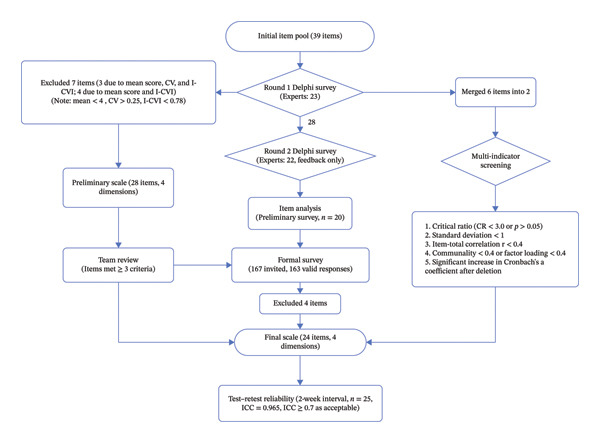
Flowchart of the scale development and validation process. Note: CR = critical ratio; CV = coefficient of variation; I‐CVI = item‐level content validity index; ICC = intraclass correlation coefficient.

The complete scale development process, including item pool construction, two rounds of expert consultation, multi‐indicator item reduction, presurvey, and formal survey, is illustrated in Figure 1.

#### 2.5.5. Validity Test

1. Content validity [12]: Six medical and nursing experts proficient in scale development and nursing management were invited to evaluate the content validity and nursing management applicability of the scale by the 4‐point Lynn scale (1 point = irrelevant/inapplicable, 4 points = highly relevant/applicable). The I‐CVI and scale‐level content validity index (S‐CVI) were calculated. The acceptable criteria were set as I‐CVI ≥ 0.780 and S‐CVI ≥ 0.800 [15].
2. Construct validity: EFA was first performed to test the structural validity of the scale. The suitable criteria [18] for EFA were KMO ≥ 0.7 and a significant Bartlett’s test of sphericity (*p* < 0.05). Principal component analysis was used to extract common factors, and varimax rotation was adopted. The number of common factors was determined based on the following criteria: eigenvalue > 1, the trend of the scree plot, factor loading ≥ 0.400, and cumulative variance contribution rate ≥ 50%.

Subsequently, CFA was conducted via the Lavaan package in R language to further verify the rationality of the scale structure. During the CFA modeling process, a Heywood case occurred in the initial model, with a negative error variance estimate for Item V12. To solve this problem, the error variance of Item V12 was fixed at 0.01 to ensure the rationality of model parameter estimation. Meanwhile, according to the prompt of modification indices (MIs), a significant residual correlation was found between Item V5 and Item V8 in the Psychological Competence dimension. Through content analysis, both items focused on patients′ psychological anxiety about disease recurrence and prognosis, with a common source of measurement error. Therefore, the error terms of Item V5 and Item V8 were allowed to correlate in the model to improve the model fit. The CFA model modifications, including fixing the variance of Item V12 at 0.01 and correlating the error terms of Items V5 and V8, were conducted based on both theoretical rationality and statistical MIs.

The error correlation between V5 (“I have made good psychological preparation for discharge home”) and V8 (“I feel psychologically relaxed about going home”) was theoretically reasonable, since both items belong to the psychological readiness dimension and measure highly similar emotional states related to discharge.

Fixing the variance of Item V12 (“I can control my emotions”) was consistent with the homogeneous response pattern of allo‐HSCT patients, as most patients tend to report positive emotional regulation, resulting in low variability in responses.

Nevertheless, we still acknowledge that such post hoc adjustments may carry a potential risk of overfitting, and further independent sample validation is still needed in future studies.

The robust maximum likelihood estimation method (MLR) was used for model fitting, which has stronger robustness to the non‐normal distribution of data [19]. The evaluation criteria for a good model fit were as follows: *χ* ^2^/df between 1 and 3, GFI ≥ 0.90, AGFI ≥ 0.85, NFI ≥ 0.90, CFI ≥ 0.90, and RMSEA ≤ 0.08 [20].

#### 2.5.6. Reliability Test

Cronbach’s α coefficient and split‐half reliability were used to evaluate the internal consistency of the scale, and test–retest reliability was adopted to assess its stability. The evaluation criteria were as follows [17, 21]: (1) Cronbach’s α coefficient ≥ 0.7 was acceptable, 0.8–0.9 was good, and ≥ 0.9 was excellent. (2) Split‐half reliability ≥ 0.7 was acceptable. (3) At least 10% of the samples were selected for retest after a 2‐week interval, and test–retest reliability ≥ 0.7 indicated good stability.

### 2.6. Statistical Analysis

SPSS 26.0 software was used for descriptive statistics, item analysis, exploratory factor analysis, and reliability testing. Confirmatory factor analysis was conducted via the Lavaan package in R language, and the results were verified by AMOS 28.0 software for robustness. The scree plot was drawn on the Heywhale platform based on the ggplot2 package in R language, and GraphPad Prism 9.0 software was used to verify the consistency of all statistical charts and tables.

Measurement data were expressed as mean ± standard deviation (*x* ± *s*), and count data were presented as frequency and constituent ratio. Experts′ enthusiasm for consultation was evaluated by the effective recovery rate of questionnaires, authority by the authority coefficient, and consistency of opinions by Kendall’s coefficient of concordance. A two‐tailed *p* < 0.05 was considered statistically significant. This scale development work adopted a cross‐sectional design, and the whole research process and manuscript presentation strictly follow the STROBE reporting standards for observational clinical research. All operations involving human participants abide by the ethical review regulations of our hospital.

## 3. Results

### 3.1. Results of Delphi Expert Consultation

#### 3.1.1. General Characteristics of Experts

A total of 22 experts completed the two rounds of consultation, coming from 10 provinces and 3 municipalities directly under the Central Government in China (Shaanxi, Hebei, Henan, Hubei, Gansu, Jiangsu, Anhui, Guangdong, Zhejiang, Beijing, Chongqing, and Shanghai). The age of the experts ranged from 30 to 59 years (42.36 ± 7.556 years), and the professional working experience was 8–40 years (21.32 ± 8.920 years). In terms of professional title, 12 experts held senior titles and 10 held intermediate titles. The professional fields included specialized clinical nursing (12 experts), nursing management (5 experts), specialized clinical medicine (1 expert), and other related fields (4 experts), covering the core areas of scale development and clinical application.

#### 3.1.2. Feedback of Expert Consultation

The effective recovery rates of the questionnaires in the two rounds of consultation were 100% (23/23) and 95.65% (22/23), respectively, indicating a high enthusiasm of experts (recovery rate ≥ 70%) [22]. The authority coefficients of the two rounds were 0.7935 and 0.7864, respectively, meeting the acceptable standard of authority (authority coefficient > 0.7) [23]. Kendall’s coefficients of concordance for importance and management applicability were 0.270 (*χ* ^2^ = 236.242, *p* < 0.001) and 0.253 (*χ* ^2^ = 218.635, *p* < 0.001) in the first round, and 0.221 (*χ* ^2^ = 131.179, *p* < 0.001) and 0.215 (*χ* ^2^ = 126.847,*p* < 0.001) in the second round. These Kendall’s coefficients of concordance, which ranged from 0.2 to 0.4, indicated moderate consistency in experts’ opinions regarding both the scientificity and management applicability of the scale. This level of agreement reflects light‐to‐moderate expert consensus, confirming that the experts’ views were generally consistent, while still leaving room for further refinement of the scale. The scale was revised according to experts′ comments and suggestions: (1) Revising dimension names: “Information Knowledge” was adjusted to “Information Knowledge and Coping Ability,” and “Adequate Support” was changed to “Social Support” to better reflect nursing management connotations. (2) Deleting 7 items that did not meet the preset elimination criteria (including low importance scores, high coefficients of variation, and low I‐CVI), with low management applicability, and merging 6 items into 3 items to improve the scale’s operability in clinical management. (3) Adding explanatory notes or examples to 4 items and adjusting the language expression of 4 items to enhance the clarity of items for both patients and nursing staff. After two rounds of consultation, the preliminary scale with 28 items across 4 dimensions was formed: Physiological Stability (5 items), Psychological Competence (9 items), Information Knowledge and Coping Ability (8 items), and Social Support (6 items).

### 3.2. Results of the Presurvey

After revising the scoring description of the scale to “completely inconsistent–completely consistent,” patients reported that the item descriptions were clear and easy to understand, and nursing staff confirmed that the scale was simple to operate and suitable for clinical management scenarios. No items were modified. The preliminary scale with 28 items across 4 dimensions was retained, and the time to complete the questionnaire was 6–15 min.

### 3.3. Results of the Formal Survey

#### 3.3.1. General Characteristics of the Study Subjects

Among the 163 patients, 85 were male (52.1%) and 78 were female (47.9%). In terms of age, 56 patients were ≤ 30 years old (34.4%), 65 were 31–50 years old (39.9%), and 42 were ≥ 51 years old (25.7%). The educational level included primary school or below (17 cases, 10.4%), junior high school (67 cases, 41.1%), senior high school or college (60 cases, 36.8%), and bachelor’s degree or above (19 cases, 11.7%). The main disease type was acute myeloid leukemia (78 cases, 47.9%). A total of 137 patients (84.0%) had no complications during transplantation, and 96 patients (58.9%) stayed in the transplantation ward for 31–45 days. Other general characteristics are shown in Table 1.

**TABLE 1:** General characteristics of the first allo‐HSCT patients (*N* = 163).

Items	Categories	Cases	Percentage (%)
Average monthly household income	< 2000 CNY	55	33.7
	2001–5000 CNY	65	39.9
	5001–10,000 CNY	29	17.8
	> 10,000 CNY	14	8.6

Marital status	Unmarried	47	28.8
	Married	112	68.7
	Divorced/widowed	4	2.5

Employment status	Employed	30	18.4
	Retired	22	13.5
	Unemployed/freelance	111	68.1

Medical expense payment method	Commercial insurance	4	2.5
	Employee medical insurance	50	30.7
	Resident medical insurance	65	39.9
	New rural cooperative medical insurance	34	20.9
	Self‐payment	10	6.1

Donor source	Parents	31	19.0
	Siblings	55	33.7
	Children	60	36.8
	Bone marrow bank	16	9.8
	Others	1	0.6

Presence of chronic diseases	Yes	17	10.4
	No	148	89.6

Driving time from home to hospital	< 0.5 h	28	17.2
	0.5–1 h	54	33.1
	1–2 h	63	38.7
	> 2 h	18	11.0

Length of stay in the transplantation ward	≤ 30 days	55	33.7
	31–45 days	96	58.9
	46–60 days	9	5.5
	≥ 61 days	3	1.8

Presence of complications during transplantation	Yes	26	16.0
	No	137	84.0

#### 3.3.2. Results of Item Analysis

Item 4, Item 13, Item 20, and Item 25 met at least 3 deletion criteria and were thus eliminated. Item 4 had a CR value of 2.904 (< 3), a standard deviation of 0.798 (< 1), an item‐total correlation coefficient of 0.244 (< 0.3), and a common factor variance of 0.37 (< 0.4), meeting four deletion criteria; Item 13 had a CR value of 1.025 (< 3), an item‐total correlation coefficient of 0.057 (< 0.3), and a common factor variance of 0.021 (< 0.4), meeting three deletion criteria; Item 20 had a CR value of 1.934 (< 3), an item‐total correlation coefficient of 0.169 (< 0.3), and a common factor variance of 0.037 (< 0.4), meeting three deletion criteria; Item 25 had a CR value of 2.085 (< 3), an item‐total correlation coefficient of 0.173 (< 0.3), and a common factor variance of 0.056 (< 0.4), meeting three deletion criteria. Finally, the formal scale with 24 items across 4 dimensions was formed. For the deleted items, Item 4 had a CR value of 2.904, a standard deviation of 0.798, a correlation coefficient of 0.244, and a common factor variance of 0.37; Item 13, Item 20, and Item 25 had a CR value < 3, a correlation coefficient < 0.3, and a common factor variance < 0.4. All other items met the retention criteria, and the detailed results are shown in Table 2.

**TABLE 2:** Item analysis results of the discharge readiness scale (*N* = 163).

Item No.	CR value	Standard deviation	Correlation coefficient	Common factor variance	Cronbach’s α	Unmet criteria	Remarks
1	4.466	1.213	0.364^∗∗^	0.936	0.843	0	Retained
2	4.708	1.137	0.380^∗∗^	0.851	0.842	0	Retained
3	4.923	1.152	0.379^∗∗^	0.889	0.842	0	Retained
4	2.904	0.798	0.244^∗∗^	0.37	0.845	4	Deleted
5	4.909	1.197	0.395^∗∗^	0.931	0.842	0	Retained
6	11.183	1.319	0.639^∗∗^	0.817	0.833	0	Retained
7	6.743	1.353	0.496^∗∗^	0.717	0.838	0	Retained
8	6.970	1.353	0.536^∗∗^	0.689	0.837	0	Retained
9	9.865	1.249	0.605^∗∗^	0.737	0.835	0	Retained
10	6.266	1.429	0.450^∗∗^	0.681	0.840	0	Retained
11	6.742	1.422	0.523^∗∗^	0.711	0.837	0	Retained
12	10.527	1.447	0.627^∗∗^	0.880	0.833	0	Retained
13	1.025	1.194	0.057	0.021	0.852	3	Deleted
14	10.289	1.470	0.617^∗∗^	0.958	0.834	0	Retained
15	7.281	1.401	0.555^∗∗^	0.879	0.836	0	Retained
16	6.217	1.341	0.494^∗∗^	0.721	0.838	0	Retained
17	5.763	1.382	0.466^∗∗^	0.770	0.840	0	Retained
18	6.211	1.357	0.534^∗∗^	0.788	0.837	0	Retained
19	6.714	1.355	0.540^∗∗^	0.806	0.837	0	Retained
20	1.934	1.104	0.169^∗^	0.037	0.848	3	Deleted
21	7.313	1.403	0.583^∗∗^	0.934	0.835	0	Retained
22	7.828	1.411	0.591^∗∗^	0.962	0.835	0	Retained
23	4.669	1.211	0.314^∗∗^	0.902	0.844	0	Retained
24	5.278	1.280	0.362^∗∗^	0.963	0.843	0	Retained
25	2.085	1.177	0.173^∗^	0.056	0.848	3	Deleted
26	4.587	1.125	0.316^∗∗^	0.673	0.844	0	Retained
27	4.304	1.250	0.324^∗∗^	0.842	0.844	0	Retained
28	4.294	1.269	0.313^∗∗^	0.911	0.844	0	Retained

*Note:* Cronbach’s α of the preliminary scale = 0.845. Item deletion criteria: CR < 3, standard deviation < 1, correlation coefficient < 0.3, common factor variance < 0.4, or Cronbach’s α coefficient > 0.845 after deletion.

^∗∗^ *p* < 0.01.

^∗^ *p* < 0.05 (two‐tailed).

#### 3.3.3. Results of Validity Test

##### 3.3.3.1. Content Validity

For the formal scale, the I‐CVI ranged from 0.830 to 1.000, and the S‐CVI was 0.92, all meeting the acceptable criteria, indicating that the scale has good content validity and nursing management applicability.

##### 3.3.3.2. Construct Validity

###### 3.3.3.2.1. Exploratory Factor Analysis

**FIGURE 2:**
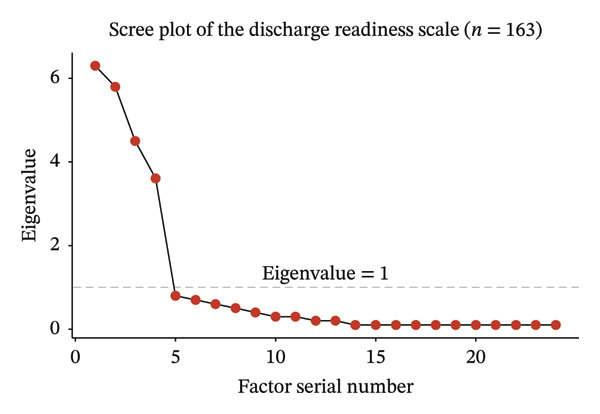
Scree plot of the discharge readiness scale (*N* = 163). Note: The abscissa represents the factor serial number, and the ordinate represents the eigenvalue. The plot was drawn on the Heywhale platform (https://www.heywhale.com/). The eigenvalues of the first four factors are all > 1, and the eigenvalues of the 5th and subsequent factors are < 1 with a gentle trend, suggesting that the division of the scale into four dimensions is rational.

① The KMO value was 0.871 with a significant Bartlett’s test of sphericity (*χ* ^2^ = 5113.590, *p* < 0.001), indicating that the scale was suitable for EFA. Four common factors were extracted, with a cumulative variance contribution rate of 83.488% (Factor 1 = 25.429%, Factor 2 = 24.412%, Factor 3 = 18.243%, Factor 4 = 15.405%). All four factors contributed substantially, with no single factor dominating. The factor loadings of all items ranged from 0.797 to 0.981 (all ≥ 0.400), and the four common factors were consistent with the original dimension design of the scale (Physiological Stability, Psychological Competence, Information Knowledge and Coping Ability, and Social Support). The detailed results are shown in Table 3.
② Scree plot: The scree plot was drawn on the Heywhale platform based on the ggplot2 package in R language (Figure 2), with eigenvalues extracted by principal component analysis and visualized. The results showed that the eigenvalues of the first four factors decreased significantly (all eigenvalues > 1), and the eigenvalues of the 5th and subsequent factors dropped rapidly to below 1 with a gentle and stable trend, further verifying that four common factors should be extracted, which was consistent with the results of EFA.

**FIGURE 3:**
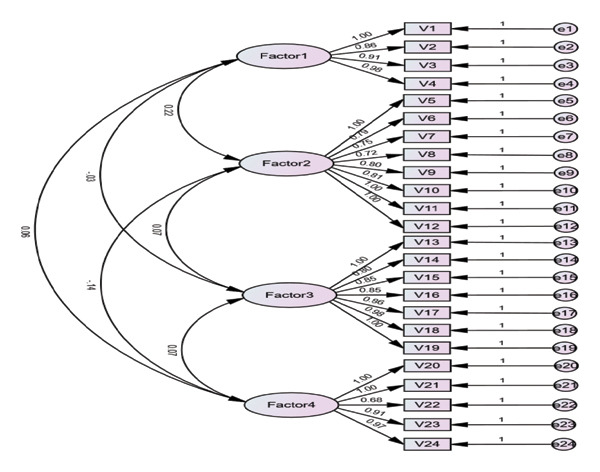
Standardized confirmatory factor analysis path diagram of the discharge readiness assessment scale. Note: The loading of the reference indicator for each factor was fixed to 1.00 to set the scale of the latent variable. Values shown are standardized factor loadings, all of which were statistically significant at *p* < 0 0.01.

(2) Confirmatory factor analysis: A 4‐dimensional structural equation model was constructed via the Lavaan package in R language. After model optimization (fixing the variance of Item V12 at 0.01 and allowing the error terms of Item V5 and Item V8 to correlate), the model showed a good fit, with all fitting indices meeting the evaluation criteria: *χ* ^2^/df = 1.795, GFI = 0.835, AGFI = 0.798, NFI = 0.918, CFI = 0.962, and RMSEA = 0.070 (90%CI: 0.059–0.081). The standardized path coefficients are presented in Figure 3. All standardized factor loadings ranged from 0.68 to 1.00, exceeding the threshold of 0.60 [18], and all were statistically significant at *p* < 0.01. The correlations between the four latent factors were low (ranging from −0.14–0.22), indicating good discriminant validity of the scale. The detailed results are shown in Tables 4 and 5, which further verified the rationality of the scale structure.

**TABLE 3:** Exploratory factor analysis results of the discharge readiness scale (*N* = 163).

Items	Factor 1 (Physiological Stability)	Factor 2 (Psychological Competence)	Factor 3 (Information Knowledge and Coping Ability)	Factor 4 (Social Support)	Communalities
Physical strength to handle daily needs after discharge	0.977	—	—	—	0.956
Energy to handle daily needs without distraction after discharge	0.925	—	—	—	0.865
Ability to complete self‐care after discharge	0.951	—	—	—	0.911
Physical changes do not affect home life	0.969	—	—	—	0.946
Psychologically prepared for discharge and returning home	—	0.894	—	—	0.817
Prepared to accept physical image changes	—	0.834	—	—	0.717
Prepared to readapt to family roles after discharge	—	0.827	—	—	0.689
Returning home makes me feel psychologically relaxed	—	0.849	—	—	0.737
Confident in the recovery of my disease	—	0.797	—	—	0.684
Worried about uncertainties after returning home	—	0.843	—	—	0.711
Hope to be closely followed up by medical staff after discharge	—	0.933	—	—	0.880
Can control my emotions well	—	0.975	—	—	0.958
Understand the complication of nursing knowledge after discharge	—	—	0.936	—	0.877
Understand symptom observation for timely treatment after discharge	—	—	0.849	—	0.723
Understand continuous medical measures after discharge	—	—	0.876	—	0.771
Understand exercise and physical activity after transplantation	—	—	0.883	—	0.788
Understand precautions for daily home life after discharge	—	—	0.899	—	0.809
Understand ways to obtain disease‐related and follow‐up information	—	—	0.966	—	0.936
Can take appropriate measures for disease‐related emergencies	—	—	0.980	—	0.962
Can get help from family and relatives after discharge	—	—	—	0.951	0.907
Can get necessary help for daily living needs after discharge	—	—	—	0.981	0.964
Have multiple ways to obtain disease‐related knowledge	—	—	—	0.811	0.672
Can contact medical staff when encountering disease‐related problems	—	—	—	0.917	0.842
Family and friends can face condition changes with me after discharge	—	—	—	0.955	0.916
Eigenvalue	6.103	5.059	4.378	3.697	—
Variance explained (%)	25.429	24.412	18.243	15.405	—

*Note:* Factor loadings ≥ 0.400 are presented; “—” indicates factor loadings < 0.400. Item deletion criteria: CR < 3, standard deviation < 1, correlation coefficient < 0.3, common factor variance < 0.4, or Cronbach’s α coefficient > 0.845 after deletion. The four factors together explained 83.488% of the total variance.

^∗∗^ *p* < 0.01.

^∗^ *p* < 0.05 (two‐tailed).

**TABLE 4:** Standardized factor loadings of confirmatory factor analysis (Lavaan package, *N* = 163).

Dimensions	Items	Unstandardized loading (est)	Standard error (se)	Z value	*p* Value	Standardized loading (std.all)
Physiological Stability	V1	1.182	0.051	23.201	< 0.001	0.979
	V2	1.813	0.060	16.887	< 0.001	0.893
	V3	1.080	0.057	19.019	< 0.001	0.941
	V4	1.158	0.057	20.224	< 0.001	0.970

Psychological Competence	V5	1.118	0.073	15.253	< 0.001	0.851
	V6	1.118	0.076	14.657	< 0.001	0.828
	V7	1.062	0.087	12.243	< 0.001	0.787
	V8	1.001	0.071	14.126	< 0.001	0.811
	V9	1.139	0.086	13.297	< 0.001	0.798
	V10	1.145	0.088	13.047	< 0.001	0.807
	V11	1.351	0.063	21.606	< 0.001	0.936
	V12	1.462	0.046	31.579	< 0.001	1.000^∗^

Information Knowledge and Coping Ability	V13	1.296	0.066	19.656	< 0.001	0.928
	V14	1.182	0.088	13.459	< 0.001	0.811
	V15	1.173	0.074	15.904	< 0.001	0.828
	V16	1.174	0.076	15.445	< 0.001	0.824
	V17	1.181	0.076	15.615	< 0.001	0.824
	V18	1.348	0.058	23.174	< 0.001	0.928
	V19	1.394	0.049	28.529	< 0.001	0.970

Social Support	V20	1.137	0.059	19.250	< 0.001	0.851
	V21	1.267	0.049	25.985	< 0.001	0.941
	V22	0.849	0.076	11.102	< 0.001	0.680
	V23	1.125	0.064	17.641	< 0.001	0.811
	V24	1.205	0.057	21.009	< 0.001	0.882

*Note:* The standardized loading of Item V12 is 1.000, which represents a Heywood case. A loading of 1.000 indicates that the item explains all variance in its factor, which may be an artifact of variance fixation during model estimation. Future studies should reexamine this item. The variance of Item V12 was fixed at 0.01 to solve the negative variance Heywood case, with a standardized loading of 1.000.

**TABLE 5:** Interdimensional correlation coefficients of the discharge readiness assessment scale (Lavaan package, *N* = 163).

Dimensions	Physiological Stability	Psychological Competence	Information Knowledge and Coping Ability	Social Support
Physiological Stability	1.000	—	—	—
Psychological Competence	0.132	1.000	—	—
Information Knowledge and Coping Ability	−0.021	0.027	1.000	—
Social Support	0.045	−0.085	0.042	1.000

*Note:* The values represent the interdimensional correlation coefficients. All absolute values of the correlation coefficients are less than 0.2, indicating good discriminant validity of the discharge readiness assessment scale.

#### 3.3.4. Results of Reliability Test

The formal scale (24 items) showed good reliability: (1) Internal consistency: The Cronbach’s α of the total scale was 0.858, and the Cronbach’s α coefficients of the four dimensions ranged from 0.955 to 0.971 (Physiological Stability = 0.971, Psychological Competence = 0.955, Information Knowledge and Coping Ability = 0.967, Social Support = 0.959). The split‐half reliability of the total scale was 0.898 (the first half = 0.908, the second half = 0.888). (2) Stability: A total of 25 patients were selected for retest after a 2‐week interval, and the test–retest reliability of the total scale was 0.965. The test–retest reliability of the four dimensions ranged from 0.888 to 0.957 (Physiological Stability = 0.934, Psychological Competence = 0.942, Information Knowledge and Coping Ability = 0.957, Social Support = 0.888).

## 4. Discussion

### 4.1. Necessity of Developing the Scale From the Perspective of Nursing Management

With the continuous development of allo‐HSCT technology, the survival rate of patients has been significantly improved, and the focus of clinical nursing has gradually shifted from improving survival rate to enhancing the quality of postdischarge rehabilitation and optimizing nursing management [2, 3]. Allo‐HSCT patients have a unique physiological and psychological state during the rehabilitation period: The long immune reconstitution cycle leads to a high risk of infection and complications; long‐term medication and physical image changes frequently contribute to negative emotions such as anxiety and depression; and the complex postdischarge care requirements put forward high demands on patients′ self‐care ability and social support [24]. These characteristics make the discharge readiness assessment of this population a key part of nursing management, and generic discharge readiness scales can no longer meet the needs of precise management [25].

As noted earlier, national healthcare priorities emphasize shortening hospital stays and rationalizing medical resource allocation [26]. Allo‐HSCT patients are often discharged early, and the lack of disease‐specific assessment tools may lead to inaccurate identification of patient needs in nursing management, inappropriate intervention measures, increased readmission rates, and waste of medical resources [25, 27, 28]. Generic discharge readiness scales fail to capture allo‐HSCT‐specific needs, particularly in areas such as GVHD monitoring, immunosuppressive medication management, and infection prevention—that are irrelevant to autologous HSCT recipients, thereby ensuring that nursing management decisions are based on allo‐HSCT‐specific rather than generic assessments. This study developed a discharge readiness assessment scale specifically for patients undergoing their first allo‐HSCT. The scale addresses key limitations of existing generic tools and may provide a useful reference for targeted discharge planning and nursing management in this population. Comparing allo‐HSCT with other complex conditions and generic scales further contextualizes its unique discharge needs. Unlike patients with heart failure or chronic obstructive pulmonary disease (COPD), whose discharge planning focuses primarily on medication adherence and symptom management for stable chronic illness [29], allo‐HSCT patients face a post‐transplant trajectory defined by profound immunosuppression, life‐threatening infection risks, and acute/chronic GVHD, requiring specialized competencies not captured in general care frameworks [24]. As noted in the Introduction, widely used generic scales such as the RHDS fail to capture allo‐HSCT‐specific needs [25, 30]. The present scale could partially fill this research deficiency, with detailed elaborations provided in Section 4.2.

### 4.2. Scientificity and Applicability of the Scale in Nursing Management

This study strictly followed the internationally recognized operational principles and methodological norms for scale development [12] and fully considered the practical needs of nursing management in the whole process, ensuring the scientificity and applicability of the scale. The scale’s rigor is strengthened by its targeted design, which addresses two key limitations of the RHDS—the most widely used generic discharge readiness scale [25]. First, the RHDS includes items irrelevant to allo‐HSCT patients (e.g., wound care, cough/sputum management) while lacking transplant‐specific recovery content. Second, evidence from transplant‐specific research confirms that allo‐HSCT patients face unique post‐transplant needs—including immunosuppressant medication safety, infection prevention, and recognition of GVHD symptoms—that are not adequately captured by the RHDS [30]. Our scale overcomes these gaps with tailored items, enabling a more accurate, comprehensive assessment for this population. First, the scale development was based on Galvin’s [8] operational definition of discharge readiness and the nursing management framework proposed by Weiss et al. [14], combined with a systematic literature review and clinical nursing management practice of allo‐HSCT. The research group included nursing management experts and clinical nurses, ensuring that the scale was both theoretically rigorous and practically operable. The Delphi expert consultation invited 22 senior experts covering clinical nursing, nursing management, and other fields and added the evaluation dimension of “nursing management applicability” on the basis of importance evaluation. The highly effective recovery rate, authority coefficient, and consistent Kendall’s coefficient of concordance indicated that the experts had a high recognition of the scale’s scientificity and management value, which ensured the scale’s applicability in clinical nursing management [23, 31].

Second, multiple comprehensive methods were used for item screening and validity testing to avoid the limitations of a single method. Item analysis adopted five methods, including critical value analysis and correlation analysis, and finally, 24 items with good discrimination and management applicability were retained. For the validity test, the content validity (I‐CVI: 0.830–1.000, S‐CVI: 0.92) of the scale was excellent, indicating that the scale items were closely related to the discharge readiness of allo‐HSCT patients and could meet the needs of nursing management. EFA extracted four common factors with a cumulative variance contribution rate of 83.488%, and the scree plot further verified the rationality of the four‐dimensional structure. CFA was conducted via the Lavaan package in R language, and the model was optimized with theoretical justification for the Heywood case and residual correlation. The optimized model showed a good fit [20], and the absolute values of interdimensional correlation coefficients were all less than 0.2, indicating a good construct validity and discriminant validity of the scale [32]. In addition, the dual software verification (Lavaan and AMOS) further ensured the robustness of the CFA results.

Finally, the scale had excellent reliability, with the Cronbach’s α of the total scale of 0.858 and the Cronbach’s α coefficients of all dimensions above 0.955, indicating a good internal consistency. The split‐half reliability (0.898) and test–retest reliability (0.965) of the total scale were far higher than the acceptable standards [21], suggesting that the scale has good stability and can be used for repeated evaluation in nursing management practice.

### 4.3. Application Value of the Scale in Nursing Management

The scale developed in this study has important application value in the clinical nursing management of allo‐HSCT patients. First, it can be used as a standardized assessment tool to help nursing managers quickly and accurately identify the discharge readiness of patients, especially high‐risk groups with low scores in Physiological Stability or Social Support dimensions, so as to formulate targeted intervention plans and improve the individualization of discharge care. Second, the scale can provide a unified evaluation standard for nursing management, realizing the standardized assessment of discharge readiness of allo‐HSCT patients, which is conducive to the quality control and continuous improvement of nursing work. Third, the scale is concise and easy to administer, taking only 6–15 min to complete, making it feasible for use in busy clinical settings. Furthermore, the multidimensional design allows for targeted, precision nursing interventions tailored to individual patient needs. For instance, a patient scoring low on the Social Support dimension could be referred to a hospital social worker for counseling or enrolled in a peer support group before discharge, strategies shown to improve psychosocial outcomes and post‐transplant survival in allo‐HSCT patients [33].

### 4.4. Limitations and Prospects

This study has certain limitations. First, the presurvey was primarily designed to assess item clarity, patient understanding, and clinical feasibility, rather than to evaluate the scale’s reliability. Therefore, reliability analyses were not performed in this phase, and only the formal survey data were used to determine the scale’s psychometric properties. Second, a Heywood case was observed for Item V12 in the CFA, which may be an artifact of variance fixation; this item will be further evaluated in future studies. Third, although the actual sample size (*N* = 163) met the minimum requirement for scale development and was within the precalculated range (154–336), it was closer to the lower bound and did not reach the ideal sample size for CFA (10–20 times the number of items), which may have influenced the stability of the factor structure and the generalizability of the findings to the broader allo‐HSCT population. Fourth, the study adopted convenience sampling and only selected patients from a single Grade III Class A hospital in Xuzhou, with a single sample source and limited representativeness. The applicability of the scale in patients from different regions, different levels of hospitals, and different disease severities needs to be further verified in nursing management practice. Fifth, this study only tested the psychometric properties and management applicability of the scale and did not further analyze the correlation between the scale scores and key management outcomes such as postdischarge readmission rate, complication rate, and nursing satisfaction, which weakens the clinical management predictive value of the scale. Sixth, all data in this study were collected through patient self‐report questionnaires, which may introduce self‐report bias, including social desirability response bias or recall bias, potentially affecting the accuracy of discharge readiness assessments to some extent. Future studies could incorporate multisource assessment methods, such as combining self‐report scales with objective clinical indicators or nurse‐rated evaluations, to reduce such bias.

In future research, multicenter, large‐sample studies will be carried out to include patients from different regions and different levels of hospitals to further verify the reliability and validity of the scale and improve its external validity in nursing management. In addition, longitudinal follow‐up studies will be conducted to explore the correlation between discharge readiness scores and postdischarge rehabilitation outcomes and management indicators, clarify the clinical management predictive value of the scale, and provide a more scientific basis for the formulation of personalized discharge nursing intervention plans for allo‐HSCT patients. At the same time, the scale will be further revised and improved according to clinical nursing management practice and research results to enhance its operability and applicability. Additionally, future work should explore integrating this scale into electronic health records to trigger automated discharge planning protocols, allowing real‐time identification of high‐risk patients and enabling timely, targeted nursing interventions.

## 5. Conclusion

The discharge readiness assessment scale developed in this study for patients undergoing the first allo‐HSCT consists of 24 items across 4 dimensions (Physiological Stability, Psychological Competence, Information Knowledge and Coping Ability, and Social Support). The scale has good reliability and validity, with a rational dimension structure verified by both EFA and CFA. It can be used as a specific and scientific tool for evaluating the discharge readiness of patients undergoing the first allo‐HSCT in clinical nursing practice and can provide a basis for clinical targeted discharge health guidance and improvement of postdischarge rehabilitation quality.

## 6. Implications for Nursing Management

This validated discharge readiness scale provides nursing managers with a standardized, disease‐specific tool to assess patients undergoing the first allo‐HSCT. It enables early identification of patients at high risk of poor postdischarge outcomes, such as those with low physiological stability or inadequate social support. By stratifying patients based on their readiness scores, nursing managers can allocate limited resources more efficiently, design targeted discharge education and follow‐up plans, and reduce unnecessary hospital readmissions.

## Author Contributions

Jiao Zhao took charge of the overall research framework design, scale item compilation, literature retrieval, statistical analysis, original manuscript drafting, and repeated revisions, acting as the primary corresponding author. The other two postgraduate authors were responsible for data entry, data sorting, and standard training for clinical staff regarding questionnaire implementation. Four experienced clinical specialist nurses participated in questionnaire collection and provided valuable clinical suggestions for item modification and scale optimization, which qualified them for authorship. One additional clinical nurse only assisted with routine questionnaire distribution, and her support is gratefully acknowledged below. Ms. Feng, head nurse of the Allogeneic Hematopoietic Stem Cell Transplantation Center, provided overall methodological guidance, supervised the entire scale development procedure, organized expert consultations, and revised multiple versions of the manuscript, serving as the co‐corresponding author.

## Funding

This work was supported by the Xuzhou Key Research and Development Program (Social Development) Medical and Health General Project under Grant No. KC22251.

## Disclosure

All authors have read and approved the final submitted version.

## Ethics Statement

This study was approved by the Ethics Committee of a Grade III Class A hospital in Xuzhou (Approval No. XYFY2023‐KL215‐01).

## Conflicts of Interest

The authors declare no conflicts of interest.

## Data Availability

Original scale items, participants’ demographic records, and confirmatory factor analysis datasets collected in this research cannot be shared publicly. This restriction follows our hospital’s institutional review board rules, aiming to safeguard the personal privacy of patients who received allogeneic hematopoietic stem cell transplantation. Researchers who require deidentified analytical data may send a formal written application to the corresponding author to gain access.
